# The Impact of Shorter, More Frequent Outdoor Play Periods on Preschoolers’ Physical Activity during Childcare: A Cluster Randomized Controlled Trial

**DOI:** 10.3390/ijerph16214126

**Published:** 2019-10-26

**Authors:** Molly Driediger, Stephanie Truelove, Andrew M. Johnson, Leigh M. Vanderloo, Brian W. Timmons, Shauna M. Burke, Jennifer D. Irwin, Patricia Tucker

**Affiliations:** 1 School of Occupational Therapy, Faculty of Health Sciences, Western University, 1201 Western Road, Elborn College, London, ON N6G 1H1, Canada; mdriedig@uwo.ca; 2 Health and Rehabilitation Sciences, Faculty of Health Sciences, Western University, London, ON N6H 4B1, Canada; struelo2@uwo.ca; 3 School of Health Studies, Faculty of Health Sciences, Western University, London, ON N6H 4B1, Canada; ajohnson@uwo.ca (A.M.J.); sburke9@uwo.ca (S.M.B.); jenirwin@uwo.ca (J.D.I.); 4 Child Health & Evaluative Sciences, The Hospital for Sick Children, Toronto, ON M5G 0A4, Canada; leigh.vanderloo@sickkids.ca; 5 Child Health & Exercise Medicine Program, McMaster University, Hamilton, ON L8N 3Z5, Canada; timmonbw@mcmaster.ca

**Keywords:** physical activity, preschooler, childcare, outdoor time, intervention, sedentary time

## Abstract

Children’s physical activity levels are higher at the start of outdoor playtime, which suggests that shorter, more frequent play periods might result in greater amounts of daily physical activity. In this extension of the Supporting Physical Activity in the Childcare Environment (SPACE) cluster randomized controlled trial, we explored the impact of four 30-min daily outdoor unstructured play periods on preschoolers’ moderate-to-vigorous-intensity physical activity (MVPA). Experimental childcare centres (n = 6) implemented four 30-min daily outdoor playtimes for 8 weeks, while control centres (n = 6) maintained their two 60-min outdoor sessions. Actical™ accelerometers were used to measure preschoolers’ physical activity pre- and post-intervention for 5 days during childcare hours. Linear mixed effects models were used to determine the impact of the intervention on preschoolers’ MVPA. Of the 185 preschoolers enrolled (54.20% female; mean age = 39.90 months, SD = 7.24), 127 (65 experimental and 62 control) were included in the analysis (30% and 9% loss to follow-up for experimental and control group preschoolers, respectively). No significant differences in MVPA were observed between groups over time (*p* = 0.36). Preschoolers’ MVPA did not improve after the introduction of shorter outdoor play periods. The loss of data due to wear time noncompliance and participant attrition may have influenced these findings. Trial registration: ISRCTN70604107 (October 8, 2014).

## 1. Introduction

Physical activity has been shown to positively impact the health of the population during the early years [1,2] with evidence to support the maintenance of healthy weight [1]; improvements in cardiovascular and bone health [1,3]; motor development [2]; social, cognitive, and emotional development [4]; and learning outcomes [5]. Further, the accumulation of total physical activity (TPA), inclusive of moderate-to-vigorous-intensity physical activity (MVPA), is associated with greater health benefits for young children [2]. In light of this research, Canadian, Australian, and World Health Organization (WHO) physical activity guidelines emphasize the value of MVPA for children over 3 years of age, suggesting that at least 60 min of the recommended daily 180 min of physical activity should be MVPA [6,7,8]. 

Many Canadian children under age six (60%) are spending a substantial portion of their week in childcare [9,10], and low levels of physical activity and high levels of sedentary time have been reported in this environment [11,12]. Specifically, children have been shown to spend as little as 1.54 min/h engaged in MVPA and approximately 41 min/h being sedentary [13]. Children of this age are influenced by the adults in their life, and In the childcare setting this includes early childhood educators. Therefore, evidence-based approaches for improving physical activity and for reducing sedentary time in childcare are critical to provide children with a solid foundation for healthy development, and including early childhood educators in these interventions is important to create a supportive environment where physical activity is built into the programming [14].

It is well recognized that children are most active when they are outdoors; [15,16,17] as such, childcare interventions that target increased outdoor time have proven effective in supporting children’s physical activity [18,19,20]. During childcare hours, children have been shown to engage in as much as 10 times more MVPA outdoors compared to indoors, i.e., 5.03 min/h versus 0.54 min/h, respectively [17]. However, a recent systematic review of the literature on children’s physical activity in childcare demonstrated that, while preschoolers spend anywhere from 6.70% to 43.00% of their time outdoors engaged in MVPA, much of this time remains sedentary (ranging from 23.2% to 63.5%) [21]. Further, a meta-analysis of studies that synthesized accelerometer-derived activity levels among preschoolers in childcare concluded that children spent an average of only 14% (10.35–17.63; 95% CI) of their time outside in MVPA [21]. Based on evidence that demonstrates that preschoolers tend to engage in higher intensity physical activity as they initiate outdoor play (i.e., in the first 10–15 min outside [16,22,23,24]), researchers have proposed that shorter, more frequent outdoor affordances may possess greater potential to improve children’s MVPA levels [16,20,25]. 

The *Supporting Physical Activity in the Childcare Environment* (SPACE) study, a cluster randomized control trial, was a multicomponent physical activity intervention implemented in 11 centre-based childcare centres in London, Canada [20,26]. Grounded in the ecological perspective of health promotion, which emphasizes the important influence that environments have on health behaviours, this intervention sought to modify the childcare setting to make it more conducive to preschoolers’ physical activity. The combination of physical activity training for childcare providers, the provision of portable play equipment, and an 8-week modified outdoor free play schedule (four 30-min versus the standard two 60-min periods) resulted in an improvement in minutes/hour of MVPA and TPA and reduction in sedentary time of preschoolers from baseline (5.38, 27.02, and 32.98 min/h, respectively) to immediately post-intervention (7.05, 28.89, and 31.11 min/h, respectively; 8 weeks) [20]. However, the noted improvements in physical activity were not sustained at 6- and 12-months post-intervention once the modified outdoor schedule was discontinued. Consequently, our team hypothesized that the shorter, more frequent bouts of outdoor play may have been responsible for the increase in physical activity [20]. Examining the outdoor play schedule in isolation (i.e., without physical activity training for childcare providers or the provision of equipment) is an important next step in this area of research. Given this intervention component is also the most cost-effective and sustainable element of the SPACE trial, it could also prove to be a feasible and promising approach for childcare centres to adopt in an effort to enhance children’s physical activity levels. 

The primary purpose of the SPACE extension was to replicate the modified outdoor schedule of the SPACE intervention to examine the independent impact of four 30-min daily outdoor unstructured play periods on the MVPA of preschoolers in centre-based childcare. The secondary outcome variables included light physical activity (LPA), TPA, and sedentary time. To provide context to implementation fidelity and feasibility, adherence to the 8-week delivery of four 30-min daily outdoor sessions was explored. It was hypothesized that children from centres that implemented the SPACE extension would exhibit higher levels of MVPA, LPA, and TPA and less sedentary time compared to children enrolled in centres that maintained standard programming. 

## 2. Materials and Methods

### 2.1. Study Design

As in the original SPACE study [26], a single-blind parallel cluster randomized controlled trial (RCT) was employed which conforms to the Consolidated Standards of Reporting Trials (CONSORT) statement [27]. A double-blind study was not possible as participants were aware of their group assignment. The study was approved by the Western University Research Ethics Board (REB # 105779).

### 2.2. Recruitment and Participants

**Figure 1:**
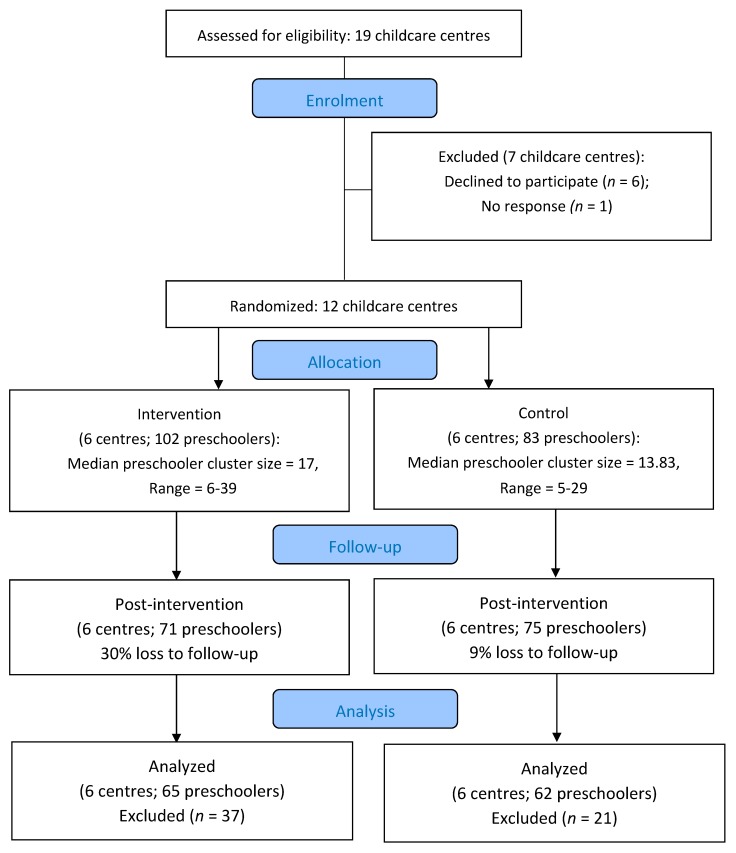
*CONSORT Flow Diagram of the SPACE Study Extension Participation Note*. No centres were lost to follow-up or dropped out of the intervention. Preschoolers were excluded due to: inadequate wear-time (*n* = 30), absent during data collection (*n* = 12), withdrawal from childcare (*n* = 14), or lost device (*n* = 2).

Eligible childcare facilities (i.e., licensed English-speaking centres with one or more preschool classrooms that did not participate in the original SPACE study [20,26]) in London, Ontario were randomly selected using a computer-generated random number list. Following written consent from 12 directors and their preschool staff, parents/guardians provided written consent for their child to participate. Centres (i.e., clusters), rather than preschoolers, were randomly assigned to receive the intervention (i.e., experimental condition; n = 6) or to maintain their usual daily programming (i.e., control condition; n = 6). Sampling was conducted at the level of the centre as it is not feasible to implement the modified outdoor playtime for some children and not others (which would be required if preschoolers were randomly assigned). A blocked randomization procedure with a ratio of 1:1 was employed to allocate centres to the experimental or control groups. All male and female preschoolers (2.5–4 years of age at baseline) with English-speaking parents from participating childcare centres were invited to participate; as such, all classes from agreeing centres participated. Childcare centre and preschooler participation for this study are reported in Figure 1. Recruitment was conducted by the project coordinator (M.D.).

### 2.3. Intervention 

Delivered by childcare providers, the 8-week intervention was implemented in childcare centre classrooms from May to August of 2017. The intervention replicated the outdoor component of the original SPACE study [20,26] in that the total time spent outdoors each day conformed to Ontario’s requisite outdoor time (i.e., 120 min) but was divided into more frequent and shorter sessions (i.e., four 30-min rather than two 60-min periods). Unstructured (i.e., child-directed) physical activity was encouraged during outdoor time. The control group maintained their typical schedule of two 60-min outdoor periods per day.

### 2.4. Data Collection

Two trained research assistants conducted all anthropometric assessments (inter-rater reliability, *r* = 98.50%) and circulated questionnaires and accelerometers at baseline (i.e., week 0) and immediately post-intervention (i.e., week 8) in both control and experimental centres. Research assistants remained blind to group assignment until post-intervention measures were complete. A change in MVPA was not sustained at 6- and 12-month follow-up in the original SPACE study [20]; as such, follow-up measurements were not conducted in the present study. 

### 2.5. Sample Size

A 2-group design required 30 participants per group assuming a small to moderate effect, a power level of 0.80, and an alpha of 0.05. Childcare centres were targeted as units (clusters); therefore, the sample size was adjusted to account for the clustering effect, where D = design effect; k = anticipated cluster size (class size in this case); and p = the intra-cluster correlation coefficient, a measure of the degree of homogeneity among cluster subjects for a particular outcome investigated (D = 1 + (k − 1) × p = 1 + (16 − 1) × (0.05) = 1.75). Given the lack of published literature related to the intra-cluster correlation (p) of PA (physical activity) among preschoolers, we used 0.05. Therefore, the design effect for an average cluster size of 16 children is 1 + 0.05 × (16 − 1) = 1.75. Thus, the sample size of each group was inflated to 30 × 1.75 = 52.5. Therefore, a total sample size of 105 preschoolers was targeted.

### 2.6. Tools 

*Accelerometers.* All participating preschoolers’ physical activity (MVPA, LPA, and TPA) and sedentary time were objectively assessed using Actical ™ accelerometers (Z and B series; Phillips Respironics, Bend, Oregon) for five consecutive days (Monday to Friday) during childcare hours at baseline and post-intervention. Preschoolers provided verbal assent to wear the accelerometers that were fixed securely to an elastic waistband and positioned on their right hip. Childcare providers were instructed to fit each participating preschooler with an accelerometer as soon as they arrived at childcare and to remove the devices at the end of each day. A daily record of wear time (i.e., when the device was put on and taken off for the day and any time it was removed) was completed by the providers. 

Accelerometry data were interpreted with Adolph and colleagues’ cut-points [28] as in the original SPACE study [20] and were used to delineate the different activity intensities: MVPA ≥ 287.5 counts·15 s^−1^·epoch^−1^; LPA ≥ 25 ≤ 287.25 counts·15 s^−1^·epoch^−1^; and TPA ≥ 25 counts·15 s^−1^·epoch^−1^. Wong et al.’s sedentary cut-points were also employed: ≤24.75 counts·15 s^−1^ [29]. Non-wear time was defined as more than 20 min of consecutive zeros [30,31]. For MVPA, LPA, TPA, and sedentary time, rates were calculated and values are reported in minutes/hour to account for variation in length of childcare day and wear time. 

*Demographic Questionnaire.* This questionnaire captured the child’s age, sex, ethnicity, annual family income, education level of the parent/guardian, and family situation. Childcare providers reported their age, sex, ethnicity, and level of education.

*Anthropometric Measures.* Anthropometric measures, including the preschoolers’ height (using a Seca 214 “Road Rod” Portable Stadiometer; nearest 0.1 cm), weight (using a Tanita 700-TBF300GS Body Fat Analyzer with Goal Setter scale; nearest 0.1 kg), and waist circumference (using a measuring tape; nearest 0.1 cm) were measured by two trained research assistants at baseline and post-intervention to calculate each child’s BMI percentile. Children removed their shoes and any clothing that would have prevented accurate measures.

*Outdoor Play Log.* During the 8-week intervention, childcare providers from the experimental and control conditions completed a daily outdoor play log. Researchers distributed one log to each participating preschool classroom after baseline measurements were complete. Developed for the original SPACE study and revised for the extension to capture the number of children who went outside, the outdoor play log included a record of the frequency, duration, and timing of each outdoor period [26,32]. Staff also indicated the reason an outdoor period may not have been possible from a list of options including weather, field trip, inadequate staff-to-child ratios, or other. 

*Program Evaluation Survey.* At the conclusion of the intervention, childcare providers in the experimental group completed a version of the SPACE program evaluation survey [32], revised to reflect the modified outdoor schedule only (i.e., education and equipment items were removed). Providers were asked to rate on a 5-point Likert scale the ease of implementation, their receptivity to the program, how effective they perceived the intervention to be at increasing children’s physical activity, their own enjoyment, their perceptions of the children’s enjoyment, and the likelihood that they would continue to implement the outdoor schedule after the intervention concluded. Three open-ended questions involved written responses to the challenges faced, solutions used to overcome these challenges, and providers’ overall experience with the intervention.

### 2.7. Data Analysis

The impact of the intervention on preschoolers’ activity levels was evaluated using linear mixed effects models for the primary (i.e., MVPA) and secondary outcome variables (i.e., LPA, TPA, and sedentary time) with group (experimental versus control) and time (baseline versus post-intervention) entered as fixed effects. Three separate models (i.e., null, main effects only, and interaction) were hierarchically tested for each outcome in order to distinguish the model-of-best-fit for the data. The null model consisted of the dependent variables (i.e., group assignment and time) predicted by random error as well as the variability associated with the different childcare centres; the main effects model tested the ability of group assignment and time separately to predict physical activity at each level, and the interaction model accounted for differential effects of group across time. Only preschoolers with two or more “valid days” (i.e., ≥5 h of wear time) at baseline were included in the analysis. All statistical analyses were performed in R [33] with linear mixed effects analyses conducted using the Ime4 [34] and ImerTest [35] packages.

Intervention implementation data documented in the outdoor play log of experimental classrooms were explored via descriptive statistics. Frequencies and percentage scores for the total number of outdoor sessions offered, the number of days that four outdoor periods were provided, the number of outdoor periods that met the 30-min time requirement, and the reasons that outdoor periods were missed were calculated. Means and standard deviations were calculated for outdoor period duration, for number of children who received outdoor time, and for items on the program evaluation survey. Responses to open-ended program evaluation survey questions were manually coded into themes and analyzed by question using a combination of inductive and deductive content analyses [36].

## 3. Results

### 3.1. Sample Description

From the 12 participating childcare centres, 185 preschoolers (54.20% female; *M*_age_ = 39.90 months; and 55.14% experimental and 44.86% control) enrolled in the study (centre and preschooler recruitment rate of 66.66% and 74.30%, respectively). After application of wear time parameters, 127 participants were retained for analyses (AVG wear time = 384.26 min/day (SD = 46.14); AVG valid days = 3.39 (SD = 1.12)). Preschoolers were largely Caucasian (70.00%) and spent ≥30 h per week in childcare (*n* = 78; 60.00%). See Table 1 for preschoolers’ and childcare providers’ demographic information.

### 3.2. MVPA

Means and standard deviations for MVPA are reported by group assignment and measurement time in Table 2. The results of the linear mixed effects model demonstrated no significant main effect or interaction effect for MVPA (α = 0.05; see Table 3).

### 3.3. Secondary Outcomes

Means and standard deviations for LPA, TPA, and sedentary time organized by group assignment and measurement time are presented in Table 2. The results of the linear mixed effects model are presented in Table 3. Results showed that, for LPA, TPA, and sedentary time, neither the main effect nor the interaction effect were statistically significant (α = 0.05).

### 3.4. Intervention Implementation and Evaluation

Frequencies and percentages for outdoor periods offered, days when four outdoor sessions were provided, outdoor periods that met the 30-min time requirement, number of periods missed due to weather, and number of children per period are presented by centre and classroom in Table 4. Mean number of minutes and standard deviations of outdoor sessions are also presented.

Childcare providers from experimental centres reported delivering 81% of total outdoor sessions, and 61% achieved the daily provision of four outdoor sessions. Of the outdoor periods offered, 75% met the 30-min outdoor time requirement with an average session length of 39.05 min (*SD* = 18.75; 156.20 min/day). Three classrooms regularly reported offering three outdoor periods per day (33–58% of total days). Across all experimental classrooms, the reasons for not going outside included field trips (*n* = 5), insufficient provider-to-child ratios (*n* = 38), and other (*n* = 86; e.g., playground renovation, animal in yard, etc.). The primary reason for a missed outdoor period was weather-related (*n* = 95), which was reported 5% of the time, primarily due to heat. Centres from the control condition reported implementing 62.67% of outdoor sessions, with an average length of 88.62 min (177.24 min/day; ranging from 31.67–163.89 min). The number of missed outdoor sessions in control centres was 27.

Childcare providers’ (*n* = 12) mean rating of the program evaluation survey items are presented in Table 5. Providers’ written responses to open-ended questions were organized into the theme of each question: challenges, solutions, and overall experience. Reported challenges included increased number of transitions, reduced time available for other aspects of the curriculum, and hesitation to interrupt children’s engagement in outdoor play. For example, one provider commented on the length of outdoor playtime: “30 min is too short of time outside”. She expressed concern for disrupting children’s activities to move indoors. Solutions that they provided included having support staff available and offering three outdoor periods rather than four. Childcare providers expressed mostly positive experiences. This is exemplified by the following quote: “The children are more aware and utilize their time better and are more involved/active”. Providers perceived the outdoor schedule to be effective in promoting physical activity.

## 4. Discussion

The present study sought to explore the independent effect of four 30-min outdoor unstructured play periods on the MVPA of preschoolers in centre-based childcare. It was hypothesized that these shorter, more frequent outdoor periods may have been the driving force behind the noted combined success of educator training, equipment, and outdoor scheduling changes that made up the original SPACE intervention [20]. However, contrary to our hypothesis, the results did not show any significant difference between groups in preschoolers’ MVPA or in the secondary outcome variables of LPA, TPA, and sedentary time. This difference may be influenced by the (unintentionally) higher rate of outdoor time (~20 min/day) afforded to young children in control centres.

The findings are not consistent with a recently conducted RCT in Australian childcare facilities that also examined the impact of short, frequent daily outdoor time (i.e., three 15-min play periods) on preschoolers’ MVPA (3–6 years; *N* = 439) [19]. The MVPA of children who received the 3-month intervention increased by approximately 5 min/day when compared to children in the control group who received the standard continuous 45-min outdoor free play session [19]. Along with four times the sample size, the brief 15-min outdoor bout implemented by Razak et al. may have been more conducive to higher levels of physical activity, and the longer implementation (3 months) may have contributed to its success. Razak et al. also indicated, anecdotally, that 80% (4 of 5) of centres continued to implement the intervention after the study ended [19]. In the SPACE study, including the current extension study, childcare providers discontinued the modified outdoor schedule as soon as it was no longer required [32]. This cessation may have been attributed to the challenges noted with increased transitions and may have been exacerbated by approaching cold Canadian weather. Additional research is needed to identify the number and duration of outdoor sessions that is not only most supportive of preschoolers’ MVPA but also sustainable within existing childcare programming.

Similar to the original SPACE intervention, childcare providers in the current study reported challenges with transitioning children when implementing four 30-min outdoor playtimes [32]. In line with other studies, transitions were more difficult for children who experienced behavioural issues, and it was reported that the added transitions disrupted the fulfilment of other more academic aspects of the childcare curriculum [19,32]. Educating childcare staff on the role of physical activity in complimenting the development of intellectual skills may be essential to motivate providers to prioritize outdoor physical activity time in addition to more academic and likely sedentary pursuits. For example, outdoor free play during the preschool years has been shown to positively correlate with characteristics of young children’s (1–5 years) temperament and has been shown to improve children’s attention and behaviour years later during elementary school [37]. Additionally, outdoor childcare playgrounds that increase children’s exposure to nature and afford challenging play experiences have the potential to positively influence children’s health and to promote higher levels of physical activity [38,39]. Therefore, it is imperative that children receive multiple daily opportunities to play outside and that time outside takes precedence over or may be combined with other educational objectives to support physical activity in childcare.

Compared to the original SPACE study, the extent of implementation of the four 30-min outdoor playtimes in the SPACE extension was lower. For example, educators provided 90% of the total number of outdoor sessions in the original SPACE study and 87% of outdoor sessions met the 30-min requirement [32]. In the SPACE extension, 81% of the total outdoor periods were offered and 75% of outdoor sessions lasted for 30 min. In both samples, outdoor periods that did not meet the 30-min criteria were typically longer than 30 min. Finally, the compliance with the requisite four daily outdoor periods was lower in the extension. Surprisingly, both studies reported missed periods due to inclement weather only 5% of the time. Despite frequent intervention centre visits by the project coordinator, reduced compliance to the intervention protocol may have limited the preschoolers’ opportunities to achieve greater amounts of higher intensity physical activity.

Solutions that childcare providers in the current study noted matched those of the original SPACE study. That is, they expressed that, as a result of the mandatory programming requirements (e.g., toileting and meals), three outdoor periods may have been more feasible to implement with two in the morning and one in the late afternoon due to naptime [32]. In examining the physical activity patterns that coincide with specific activities, other researchers have found that children in childcare are most active in the morning [15]. Therefore, encouraging additional outdoor periods during the first half of the day may be more conducive to promoting physical activity in this environment and may be more feasible for childcare scheduling purposes, lending support for sustained widespread implementation. As other researchers have found three periods of outdoor free play in childcare to be effective in supporting higher intensity physical activity among young children, it is promising and substantiates the potential of this strategy to be tested on a larger scale [19].

## 5. Strengths and Limitations

Strengths of this study were the RCT design and the use of accelerometers to measure physical activity among children. Also, that the timing, duration, and number of children who experienced each of the four daily outdoor periods were assessed for the entire 8-week intervention and provides a greater understanding of intervention fidelity. However, characteristics of the childcare centres and outdoor playgrounds were not examined, and there may have been variability in the quality and presence of equipment/facilities (e.g., space, fixed play structure, natural elements, toys, etc.). The childcare centre and outdoor play environment have been shown to significantly influence children’s play experiences and levels of physical activity [40,41]. Given the insignificant results, the small sample size, and the lack of childcare centre quality measurement, future investigations of modified recess should consider characteristics of the facility’s outdoor play space.

Despite the provision of accelerometer training to childcare staff, including strategies to promote children’s motivation to wear the devices, rates of attrition and inadequate wear time were high. The summer implementation of the extension intervention may have contributed to the loss to follow-up, given that children were often absent due to vacation. Participation at post-intervention was also influenced by the unexpected early advancement of older (i.e., 3.5–4.5 years) children into school-age programs in two of the experimental centres. Given the previously demonstrated positive association between MVPA during outdoor time and children’s age [15], the loss of older children who may have contributed more substantially to higher levels of physical activity likely affected the results. Loss of participation, whether due to inadequate wear time or attrition, particularly in the intervention group, led to decreased power within the study. Additionally, summer implementation of this intervention resulted in missed outdoor play sessions due to heat.

## 6. Conclusions

The implementation of the SPACE extension did not support improved MVPA, LPA, or TPA or reduced sedentary time among preschoolers during childcare hours; however, children from the control centres received, on average, 20 more minutes per day of outdoor time, which may have influenced these results. The unexpected loss of older participants at post-intervention and the high rates of attrition experienced during physical-activity measurement may have influenced these results. Additionally, the interest and habits of childcare providers combined with the weather may have impacted the implementation of the four outdoor playtimes. Further investigation is necessary to explore the “ideal” and “feasible” combination of frequency and duration of outdoor time for young children in childcare and the sustainability of a revised outdoor curriculum.

## Figures and Tables

**Table 1:** Descriptive characteristics of enrolled preschoolers and experimental childcare providers.

Variable	Control (*n* = 83)	Experimental (*n* = 102)	Childcare Providers (*n* = 17)
Age, months (children)/years (adults), *M* (*SD*)	37.75 (6.25)	41.98 (7.57)	36.20 (14.40)
Sex, *n* (male/female)	28/38	32/33	0/10
BMI Percentiles, *M* (*SD*)	52.92 (21.37)	56.09 (31.65)	
Hours in Childcare			
<10	4	5	
10–19	8	7	
20–29	10	18	
30+	43	35	
Ethnicity			
Caucasian	51	40	9
African Canadian	1	3	0
Native/Aboriginal	0	5	0
Arab	1	7	0
Latin-American	3	1	0
Asian	2	1	0
Other	6	7	1
Family Income			
<$20,000	1	17	
$20,000–$59,999	14	20	
$60,000–$99,999	12	8	
$100,000–$149,999	19	10	
>$150,000	12	3	
Highest Level of Education			
Secondary	6	17	1
College	27	19	8
University	21	14	1
Graduate School	12	11	0
Family Situation			
Single Parent	14	14	
Double Parent	51	41	
Other	0	2	

*Note*. Frequencies (*n*) unless otherwise noted. Frequencies may not add up to *n*-size due to missing data.

**Table 2:** Mean (SD) in minutes/hour of preschoolers’ physical activity and sedentary time.

	MVPA		LPA		TPA		ST	
Time	Control	Exp.	Control	Exp.	Control	Exp.	Control	Exp.
Pre (Week 0)	7.06 (3.22)	7.66 (3.05)	24.29 (3.66)	23.07 (3.39)	31.34 (5.79)	30.74 (4.51)	28.65 (5.79)	29.26 (4.51)
Post (Week 8)	7.04 (3.13)	6.98 (2.97)	23.46 (3.30)	23.55 (3.51)	30.50 (4.92)	30.53 (4.86)	29.50 (4.92)	29.47 (4.86)

*Note*. *SD* = standard deviation; MVPA = moderate-to-vigorous physical activity; LPA = light physical activity; TPA = total physical activity; ST = sedentary time, Exp. = experimental condition.

**Table 3:** The effect of the SPACE extension on MVPA, LPA, TPA, and ST.

Model	MVPA			LPA			TPA			ST		
	*df*	χ^2^	*p*	*df*	χ^2^	*p*	*df*	χ^2^	*p*	*df*	χ^2^	*p*
^1^ Main effects	2	2.22	0.33	2	3.24	0.86	2	1.06	0.59	2	1.06	0.59
^2^ Interaction	3	2.76	0.43	3	3.24	0.36	3	1.54	0.67	3	1.54	0.67

*Note*. ^1^ tested against the null model; ^2^ tested against the main effects model. MVPA = moderate-to-vigorous physical activity; LPA = light physical activity; TPA = total physical activity; ST = sedentary time; df = degrees of freedom; χ^2^ = chi-square. α = 0.05.

**Table 4:** Adherence to four 30-min daily outdoor periods by experimental centres (class).

Centre (Class)	Total Number of Outdoor Sessions Offered ^†^ (%)	Number of Days with 4 Outdoor Sessions * (%)	Number of Total Sessions Lasting 30 Minutes (%)	Mean Outdoor Session Duration in Minutes (*SD*)	Number of Missed Sessions due to Weather (*n*)	Number of Kids (Median)
1(a)	90 (57.69)	15 (38.46)	86 (95.55)	30.84 (5.94)	18	7
1(b)	98 (62.82)	17 (43.59)	73 (74.49)	31.28 (9.20)	10	7
2(a)	117 (73.72)	14 (35.90)	76 (64.96)	37.36 (11.61)	9	7
2(b)	153 (98.08)	36 (92.31)	86 (56.21)	41.90 (17.93)	3	5
2(c)	154 (98.72)	37 (94.87)	96 (62.34)	41.33 (16.37)	2	4
2(d)	152 (97.44)	24 (92.31)	93 (61.18)	41.67 (17.06)	4	5
3	135 (88.82)	27 (72.97)	132(97.78)	31.76 (18.78)	17	7
4(a)	147 (96.71)	34 (91.89)	123 (83.67)	33.18 (9.96)	5	5
4(b)	144 (94.74)	33 (84.62)	138 (95.83)	31.84 (10.32)	4	6
5(a)	118 (77.63)	13 (35.14)	69 (58.47)	60.91 (46.17)	0	3.50
5(b)	83 (54.61)	3 (8.11)	53 (63.86)	46.02 (23.87)	1	3
6	107 (70.40)	14 (37.84)	88 (82.24)	40.52 (37.82)	22	8
Grand Mean	124.83	22.25	92.75	39.05	7.92	5.63

*Note*: * Total number of days possible = 39. ^†^ Total number of outdoor sessions possible = 156.

**Table 5:** Childcare providers’ mean (SD) rating of intervention feasibility, effectiveness, enjoyment, and future implementation.

Construct	Item	Mean	*SD*
Feasibility ^†^	The intervention was easy to implement.	2.83	1.15
	When first approached to participate, I was very receptive to this intervention	4.08	1.17
	The four 30-min outdoor play periods were easy to implement.	3.00	0.95
Perceived Effectiveness ^∞^	The four 30-min outdoor play periods were effective.	3.25	0.75
Educator’s Enjoyment ^±^	The four 30-min outdoor play periods were enjoyable for me.	3.00	0.74
Children’s Enjoyment ^±^	The four 30-min outdoor play periods were enjoyable for the children.	3.33	0.99
Future Implementation ^§^	Likelihood of continuing to implement the four 30-min outdoor play periods.	2.50	0.91

*Note*: Mean scored from 1 to 5; *SD* = standard deviation; Respondents were asked to rate the above statements from: ^†^: 1 (strongly disagree) to 5 (strongly agree); ^∞^: 1 (not at all effective) to 5 (extremely effective); ^±^: 1 (not at all enjoyable) to 5 (extremely enjoyable); and ^§^: 1 (not at all likely) to 5 (extremely likely).

## Abbreviations

SPACE	Supporting Physical Activity in the Childcare Environment;
MVPA	moderate-to-vigorous physical activity;
TPA	total physical activity;
WHO	World Health Organization;
LPA	light physical activity;
RCT	randomized control trial;
CONSORT	Consolidated Standards of Reporting Trials;
BMI	Body Mass Index.
